# Photonic Time Crystals and Time‐Varying Electromagnetic Metamatter: A New Direction for Ultrafast Tunable Photonic and Microwave Materials and Devices

**DOI:** 10.1002/advs.202519790

**Published:** 2026-01-26

**Authors:** Ranjan Kumar Patel, Shriram Ramanathan, Ronald P. Jenkins, Michael J. Carter

**Affiliations:** ^1^ Department of Electrical & Computer Engineering Rutgers The State University of New Jersey Piscataway New Jersey USA; ^2^ Materials and Manufacturing Directorate Air Force Research Laboratory Wright Patterson Air Force Base Dayton Ohio USA; ^3^ Core4ce Fairborn Ohio USA

**Keywords:** metamatter, metasurfaces, photonic crystals, time crystals, ultrafast

## Abstract

Spatial control of materials properties through nanostructuring and hierarchical design is an important on‐going area of materials research. Controlling physical properties in time represents a new direction to further advance the frontiers of functional materials and devices with particular attention to matter‐radiation interactions. Such time‐varying crystals and dynamic heterogeneous media can interact with radiation in an unexpected manner, resulting in emergent properties that have no static counterpart. Here, we briefly discuss theoretical and analytical techniques used to understand time‐varying electromagnetic materials and highlight novel phenomena in these systems. Further, we examine the state‐of‐the‐art numerical modeling tools and design techniques for photonic time crystals and time‐varying metamaterials and highlight the need for continued efforts to develop these tools. Representative examples from experimental studies on thin films, 2D materials, and mesoscale composites that are essential building blocks for time metamatter are then reviewed toward high‐speed modulation of optical constants. Finally, we discuss outstanding materials challenges in this emerging field and point out that collaborative cross‐disciplinary research is needed to better understand photon interactions with electrons, spin order, and the lattice.

## Graphical abstract

Photonic time crystals (PTCs) are systems in which electromagnetic parameters are modulated periodically in time, producing momentum bandgaps via temporal scattering rather than spatial Bragg processes. This review examines the theoretical frameworks, modeling, and computational tools for time‐varying media, and summarizes experimental demonstrations to date from RF to optical regimes, and highlights materials challenges and key directions for further discoveries of ultrafast time‐varying photonic systems.

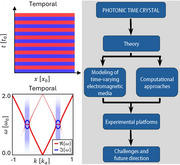

## 1 Introduction

Artificial electromagnetic materials have long garnered the interest of scientists and engineers due to their ability to pristinely control the spectral and spatial properties of propagating fields. Traditional paradigms for artificial electromagnetic materials, such as photonic crystals, metamaterials, and metasurfaces tailor the spatial dielectric properties (i.e., ϵ(r)) of an engineered material [1]. This is achieved by patterning deeply subwavelength (<λ/10, where λ is the wavelength) features, giving rise to an effective medium‐like behavior [2], or more often, by careful tailoring of the local and/or nonlocal response of subwavelength (∼λ/2) structures [3, 4, 5, 6] to achieve pristine control over the flow of light and its various degrees of freedom. Efforts to tailor the spatial dielectric properties in two‐ or three‐dimensions (2D or 3D) have led to several compelling demonstrations ranging from compact planar optical components [4, 5, 7, 8, 9], to engineered materials with giant nonlinearities [10, 11, 12] and even demonstrations of non‐reciprocal [13, 14] and non‐Hermitian performance [15].

Given the depth and breadth of research on 2D and 3D spatially inhomogeneous electromagnetic materials, it is only natural to consider extensions to a fourth dimension, *time*, and carefully tailor the time‐varying dielectric properties (i.e., ε(*t*)) of an engineered material [16]. Indeed, this area of research has seen considerable interest in the past few years, with potential applications in optical isolators [17, 18, 19, 20] and modulators [20, 21], optical parametric amplification [22, 23], correlated‐photon‐pair generation [24] and as frequency mixers [25], circulators [19, 20] or other components for optical and radiofrequency (RF) communications [26, 27, 28]. This application potential is due in large part to the programmability [26, 27], novel communication modes, such as leveraging non‐reciprocity for full‐duplex operation [26, 28], and functional improvements, such as suppressing unwanted mixing products [25], that time‐varying materials and devices offer. Beyond promising applications, time‐varying metamaterials offer a rich platform to study a wide array of interesting phenomena, most notably nonreciprocity [18, 29, 30], time‐reflection/refraction [31, 32], and transient interactions with more complex electromagnetic modalities such as surface plasmon‐polaritons and modulation of lattice properties [33, 34, 35].

To further elucidate the role of time‐varying media in nonreciprocal photonics, it is important to establish how time interfaces and photonic time crystals (PTCs) enable nonreciprocal mechanisms that are distinct from those based on spatial asymmetry or nonlinearity. In particular, temporal modulation breaks time‐translation symmetry while preserving spatial homogeneity, providing a fundamentally different pathway to nonreciprocal wave propagation. At a temporal interface, electromagnetic waves conserve momentum while undergoing frequency and amplitude conversion, enabling asymmetric mode coupling between forward and backward propagating states. This mechanism was discussed by Li et al. [36] through the temporal analogue of Faraday rotation in magnetized media, establishing nonreciprocal polarization rotation without spatial boundaries. Extending this concept, He et al. [37] showed that periodic temporal modulation in nonreciprocal photonic time crystals enables Floquet band engineering that supports momentum bandgaps, directional amplification, and enhanced temporal Faraday effects. More generally, as discussed by Mendonça [38], sequences of temporal interfaces can produce robust magneto‐optical phenomena, including Faraday and Cotton‐Mouton effects, arising from frequency shifts, amplitude imbalance, and accumulated phase differences between eigenmodes. Together, these works clarify the role of time interfaces and PTCs as powerful platforms for realizing nonreciprocal wave propagation in spatially homogeneous, time‐modulated media.

Recent efforts have also begun to clarify the similarities between time‐varying electromagnetic phenomena and traditional parametric processes [39], as well as explore their physical bounds [40, 41]. These steps are essential for grounding expectations in a field experiencing a bit of a renaissance, building on work that dates back to at least the 1950s [42, 43]. This renewed interest presents an ideal opportunity to address a wide variety of open scientific challenges. Most notably, time‐varying metamaterials and PTCs benefit from abrupt and strong modulation of the constitutive dielectric properties of materials. Realization of PTCs and achieving parametric amplification in momentum bandgaps typically requires modulation speeds on the order of a single wave cycle [39, 40]. More recent work on parametric amplification via time‐varying electromagnetic phenomena has clarified that modulation time periods only need to be faster than the propagation time, and not as fast as the period of a single wave cycle [39]. Applications such as modulators, circulators, and isolators have relaxed requirements on temporal modulation frequency, and can be realized at modulation frequencies a few orders of magnitude less than the operational frequency [18, 26, 27, 40].

This still places a significant demand, particularly at optical frequencies [44], on materials that can support large and fast changes in refractive index, especially for PTCs, where all‐optical modulation of a nonlinear material is the most reasonable means to realize a PTC. Electro‐optic modulation and other tuning approaches with more modest modulation speeds are more suitable in applications such as isolators, modulators, and circulators, owing to their previously noted relaxed modulation speed requirements. Several material platforms have been actively explored experimentally to realize time‐varying functionality across different frequency regimes, each tailored to application‐specific demands of temporal modulation amplitude and speed [45, 46, 47]. At microwave frequencies, temporally modulated transmission lines and varactor diode‐loaded circuits [48] provide a well‐controlled and experimentally accessible route to emulate PTCs [49]. At optical frequencies, the material constraints are more severe; experimental strategies rely on ultrafast nonlinear optics, epsilon near zero (ENZ) materials, and photo‐induced phase transitions to achieve index modulations approaching unity on sub‐femtosecond timescales [50, 51, 52]. Optically‐driven surface and thin‐film systems have also shown promise for ultrafast gratings and transient quasi‐BICs [35, 53, 54]. Recently, 2D materials and metasurfaces have also emerged as promising candidates for realizing photonic time crystals, owing to their tunable surface properties and compatibility with ultrafast modulation schemes [55]. Beyond these material challenges, considerable effort is also needed to improve both theoretical and numerical techniques to model time‐varying artificial electromagnetic materials [28]. Most widely available full‐wave solvers are not yet able to natively handle temporal variation in their constitutive material properties. Moreover, inter‐harmonic coupling in time‐varying materials adds a significant degree of computational cost. Several recent efforts have begun to close this gap [56, 57, 58, 59], however more effort is needed to realize computational tools that can model large‐scale complex designs, such as spatially‐ and temporally‐varying metamaterials in a rigorous and efficient manner.

Through this Perspective article, we aim to provide a brief overview of the growing field of research concerning PTCs and time‐varying metamaterials, covering theory, computational and experimental efforts, and, along the way highlight promising modeling and design techniques and materials platforms primed for further research efforts, which could help address previously mentioned challenges. We will start, in Section 2, by introducing theoretical and analytical techniques used to explore time‐varying electromagnetic media and briefly highlight novel phenomena in these systems. We will then review existing numerical modeling tools and design techniques for PTCs and time‐varying metamaterials and highlight the need for continued efforts to develop tools for design, modeling, and simulation of time‐varying optical systems. Next, in Section 3, we will provide an overview of experimental realizations of PTCs and temporal metamaterials, to date, examining various material platforms, modulation mechanisms, and key trade‐offs that define their suitability for future implementations. Finally, we will conclude this perspective with brief remarks on the challenges and promise of continued fundamental research efforts regarding PTCs and time‐varying metamaterials and the need for strong interdisciplinary efforts to help time‐varying materials reach their potential.

## 2 Theory and Modeling of Photonic Time Crystals and Time‐Varying Metamaterials

**FIGURE 1:**
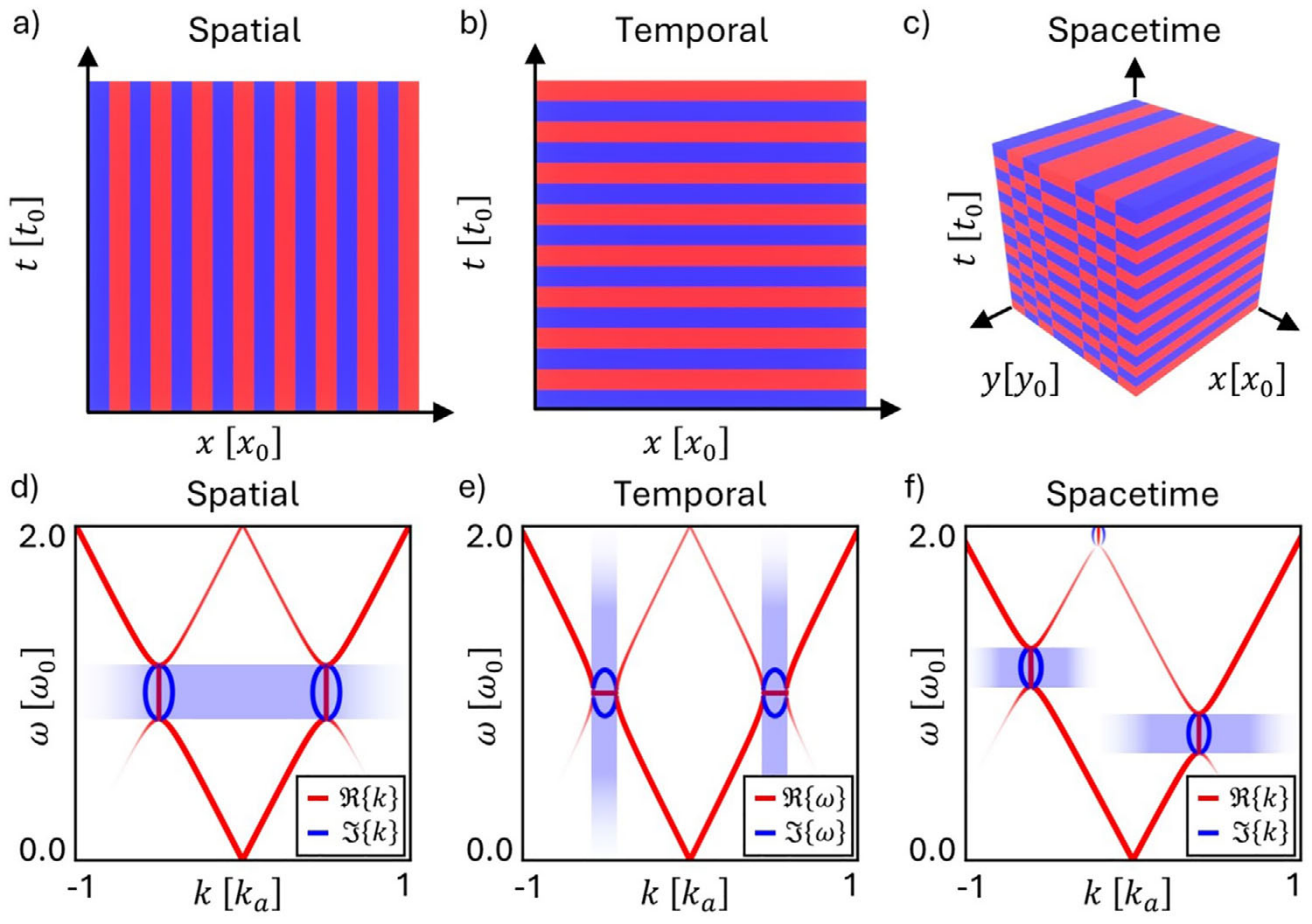
Similarities and differences between conventional photonic crystals and PTCs. (a), (b), and (c) space‐time diagrams of permittivity for photonic space crystal, time crystal, and space‐time crystal, respectively. (d), (e) and (f) dispersion diagram with band gaps for both space crystals, time crystals, and space‐time crystals, respectively.

One of the most effective strategies for analyzing PTCs is to study their behavior in the context of Bloch‐Floquet Theory [46]. Conventional photonic crystals (Figure 1a) can be shown to admit a discretized set of wavenumbers *k_n_* (momenta) called Bloch modes which arise from the lattice geometry. Any field at some frequency ω incident on the photonic crystal must be decomposed into these modes, a property readily captured in the dispersion diagrams of photonic crystals (Figure 1d). By selecting a periodic patterning of material within the crystal lattice, band gaps can be created for which the only admitted modes are evanescent, having non‐zero imaginary parts in their wavenumber. Consequently, if the materials used are lossless, then within these band gaps, the photonic crystal must act as a lossless reflector in this regime.

A direct analogy can be drawn between this linear time‐invariant (LTI) system and the behavior of PTCs, a periodic (ϵ (t)= ϵ(t+T)) linear time‐varying (LTV) system. Whereas conventional photonic crystals are periodic in space, PTCs are periodic in time (Figure 1b), leading to a Bloch–Floquet expansion of the fields, which discretizes the frequencies admitted within the PTC. When analyzed in the same framework as conventional photonic crystals, a periodic patterning of material in time will indeed create a band gap, but in momentum rather than frequency (Figure 1e). Extending this analysis, spacetime crystals (Figure 1c), we see tilted bands for traveling wave spacetime modulation (Figure 1f). This highlights a hallmark feature of time‐varying metamaterials, the ability to break Lorentz reciprocity.

Bloch‐Floquet modeling is an effective technique for computing the dispersion properties of simple bulk crystals (time‐varying or otherwise), but it cannot provide insight into the specific behavior of finite‐sized devices that incorporate PTCs. Full‐wave analysis is needed for such applications, particularly when it comes to applying more realistic engineering constraints. Numerical methods for solving LTV device scattering and modes (Table 1) are an active area of research, with the number of recent contributions growing alongside emerging interest in LTV systems. These tools come in many flavors, each suited to the needs of its class of problems. From a birds‐eye view, this new class of solvers can be slotted into several overlapping categories depending on a few key differences.

**TABLE 1:** Summary of LTV numerical methods and their properties. The solvers collected here are research codes, and so many are implemented at reduced dimensionalities (without loss of generality) to demonstrate their functionality. Direct runtime comparisons will become more relevant and readily available as these techniques mature. LTV control parameters: ε = permittivity, μ = permeability, σ = conductivity, κ = magnetic loss, *Z* = impedance, *Y* = admittance, *C* = capacitance, $\omega_{0}^{2},\;\omega_{p}^{2},\;\alpha$ are Lorentzian parameters.

Refs.	Method(s)	TD/FD	Dim.	Params	Field Format	Notes
[56, 60]	FDTD	TD	2D	ε, μ, σ	Yee Cells	Full‐wave
[61, 62]	TD‐FIT	TD	3D	ε, μ, σ, κ	Conformal Yee	Full‐wave
[64]	ODE Solve	TD	0D	ε, μ	Time‐only	PTC
[63]	TD‐MoM	TD	3D	*Z*, *Y*	Green's Function	Thin‐wire
[46, 67]	PWEM	FD	1D	ε, μ	Floquet	Eigensystem
[65]	TMM	FD	2D	ε	Floquet	Slabs
[27]	Circuit	FD	2D	*C*	Floquet	Metasurface
[59]	Circuit + MoM	FD	3D	*C*	Green's Function	Metasurface
[68]	2‐State Circuit	FD	2D	*Y*	Floquet	Metasurface
[66]	Mie Theory	FD	3D	σ	VSH	Shell
[69, 70]	FD‐MoM	FD	3D	*Z*	Green's Function	Single Load
[57]	FD‐MoM	FD	3D	*Z*	Green's Function	Multi‐load
[58]	GSTC Floquet	FD	2D	$\omega_{0}^{2},\omega_{p}^{2},\alpha$	Floquet	Metasurface

The first critical point of difference is the solver's simulation domain. LTV materials have been introduced to many time domain (TD) methods such as the finite difference time domain (FDTD) method [56, 60], the time domain finite integration technique (TD‐FIT) [61, 62], time domain method of moments (TD‐MoM) [63], and other more specialized iterative techniques [64]. Frequency domain (FD) methods have received at least equal treatment, with LTV materials being incorporated into many Floquet methods [27, 58, 59, 65], Frequency domain method of moments (FD‐MoM) [57, 59], and even Mie theory [66] to name only a few.

A second essential difference of these solvers is what parameters are temporally varied, and what level of homogenization has been applied to the LTV components involved. Many methods approximate LTV surfaces as generic sheet properties such as time‐varying conductivity [66], spacetime‐varying Lorentzian oscillators in Generalized Sheet Transition Conditions (GSTCs) [58], or as the capacitance of a homogenized metasurface element [27]. Others eschew homogenization of circuit components and simulate them directly [57, 62, 63]. Still others focus on time‐varying volumetric parameters for the materials, such as permittivity and permeability [46, 56, 61], which may be more relevant to optical frequency LTV devices.

Lastly, each method has its own strategy for representing fields depending on the formulation. MoM techniques will use various Green's functions to represent scattered fields. The Floquet methods form a set of harmonics based on their device's spatio‐temporal lattice. Most generically, FDTD and TD‐FIT both have local definitions of the field on a grid or mesh. Green's function or Floquet techniques can be particularly useful for devices that can be accurately treated as an active surface under some homogenization scheme. On the other hand, volumetric devices such as nonlinear optical media or antennas with lumped LTV components may demand full‐wave techniques for sufficient accuracy.

It might seem preferable at first glance to prioritize TD techniques for modeling time‐varying materials. Material variation is more straight‐forwardly represented in the TD, but as it turns out, the choice is not so simple. One benefit of TD solvers in LTI contexts is that they are inherently broadband and can thereby accelerate the measurement of a device's scattering over a wide frequency range. LTI devices do not scatter between frequencies, and so this property of TD solvers is purely beneficial. In contrast, FD solvers must separately simulate each discrete frequency, which may be problematic for evaluating the broadband operation of some devices. However, while correct for LTI simulation, this cost‐benefit calculus changes once time‐variation is introduced and causes coupling between harmonics. Scattering at any given frequency from an LTV device, in general, depends on the entire spectrum of the source used. Put another way, the inter‐harmonic scattering matrix will always be diagonal for LTI devices but non‐diagonal for LTV devices, meaning a single broadband TD simulation cannot be used to uniquely probe all its columns. Optimal decision making about which category of solver to use (TD or FD) for a given LTV system is therefore highly dependent on the spectral content of the source to be simulated and should be treated in conjunction with other standard considerations related to the device and its geometry.

**FIGURE 2:**
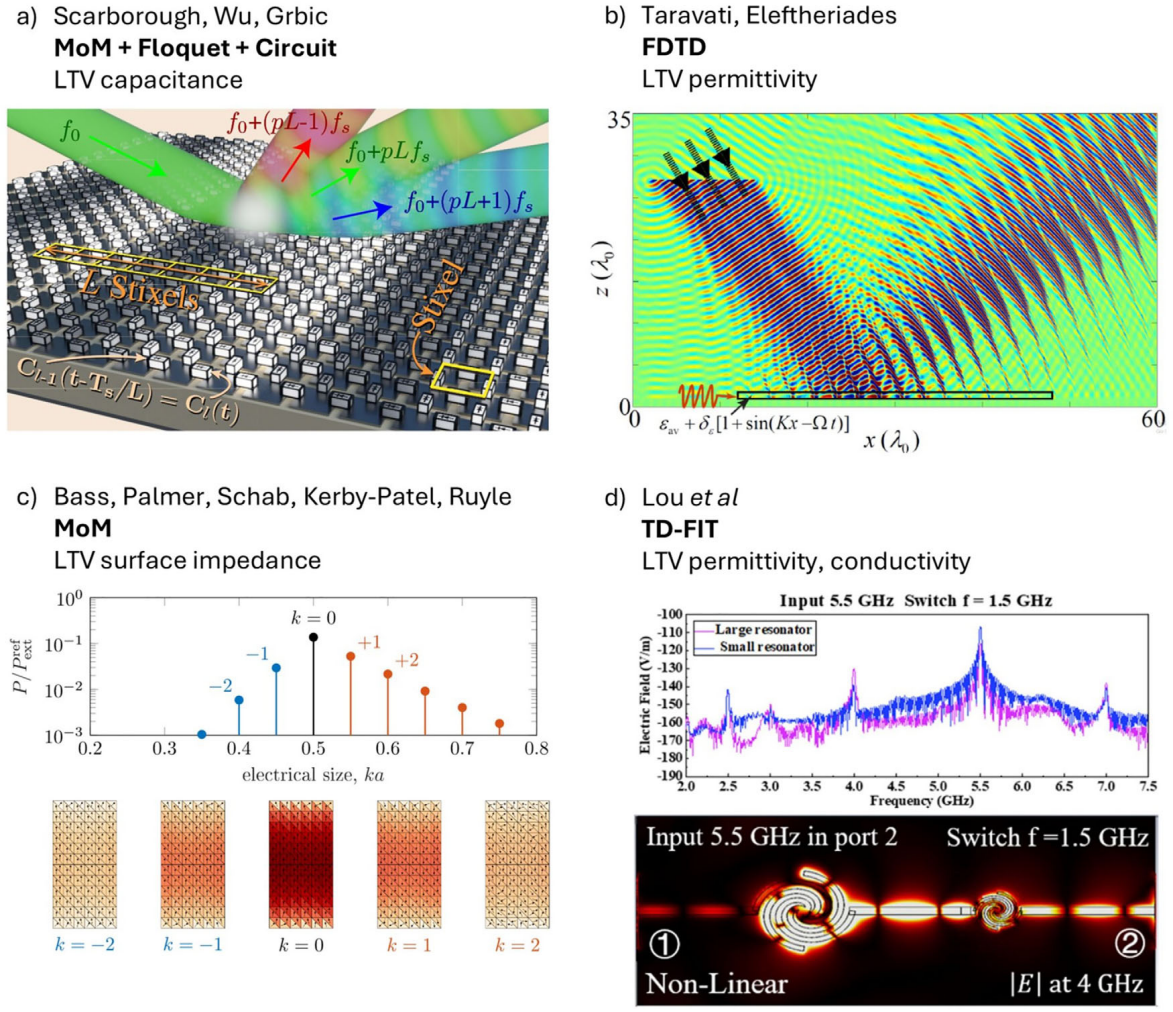
(a) Metasurface unit cells with a spacetime modulated capacitance can be solved efficiently using the interpath relation and Floquet analysis. (b) FDTD is readily augmented with time‐varying permittivity to model scattering from finite‐thickness spacetime metasurfaces. (c) Introducing time‐varying conductivity to MoM allows scattering from antenna systems that have been augmented to have a homogenized time‐varying conductivity or lumped elements. (d) TD‐FIT is a meshed (i.e., conformal) generalization of FDTD, now modified to allow time‐varying materials and boundary conditions. Panel (a) is reproduced with permission [59]. Copyright 2021, IEEE. Panel (b) is reproduced with permission [60]. Copyright 2019, American Physical Society. Panel (c) is reproduced with permission [57]. Copyright 2022, IEEE. Panel (d) is reproduced with permission [61]. Copyright 2022, MDPI.

Early efforts to develop computational tools for LTV electromagnetic systems have expanded the suite of modeling techniques relevant to PTCs and time‐varying metamaterials in both the RF and optical regimes (Figure 2). More work is needed to extend techniques demonstrated in the literature to 3D, which is certain to elevate time complexity concerns, and develop more efficient, accurate, and broadly applicable LTV modeling tools. Nevertheless, even in their nascent form, these solvers have the potential to provide numeric corroboration and direction to experimental efforts, further accelerating the exciting developments in this area.

## 3 Path Toward Experimental Realization

Having outlined the theoretical foundations and modeling approaches for PTC and time‐varying metamaterials in the previous section, we now turn to experimental efforts. While theory predicts a rich set of phenomena arising from temporal modulation, translating these concepts into physical systems requires careful material selection, understanding of underlying mechanisms, and precise control over ultrafast dynamics. In the following section, we survey the key experimental platforms developed to date, highlighting both the practical implementations and the material challenges that define the current state of the field.

As noted in Section 2, PTCs differ fundamentally from conventional spatial photonic crystals as the spatial periodicity results in frequency bandgaps due to Bragg scattering in real space, temporal periodicity induces momentum bandgaps arising from coherent interference between forward and backward propagating waves scattered in time [45, 46, 71, 72, 73, 74, 75, 76]. This time‐domain scattering leads to novel phenomena such as time‐reflection and time‐refraction, which have no direct analog in static systems. The experimental realization of PTCs remains nontrivial, largely due to the demanding requirements on modulation speed and amplitude. However, recent developments across several frequency regimes have opened promising routes for implementation and feasibility studies.

In this section, we provide a systematic overview of the experimental platforms currently explored for realizing PTCs. We begin with systems operating at radio frequencies, where PTC behavior can be emulated using transmission lines. Next, we discuss the use of metasurfaces, ultrathin and structured metamaterials as platforms for implementing time‐varying responses. Finally, we discuss efforts and material strategies to realize PTCs at optical frequencies, which present significant technical challenges. Specifically, achieving observable time‐reflections or appreciable momentum bandgaps requires modulating the refractive index of the medium by a substantial amount (typically on the order of unity) within a time scale comparable to an optical cycle, i.e., within a few femtoseconds [40, 47]. An external periodic drive is necessary to induce these time‐varying optical properties, much like the external pumping used to realize discrete time crystals [46, 72]. Such ultrafast and high‐contrast modulations are difficult to achieve, but recent progress in nonlinear optics, ENZ materials, and ultrafast pump‐probe techniques offers potential pathways.

### 3.1 PTCs in Radio Frequency Regime

Fundamental phenomena such as time‐refraction, time‐reflection, and the formation of momentum band gaps have been investigated at radio frequencies by emulating time‐varying media through temporally modulated transmission lines [49, 77, 78, 79]. Among these effects, time‐refraction is relatively straightforward to observe, as the time‐refracted wave maintains its original wave vector while propagating, experiencing only a change in frequency due to the temporal discontinuity in the medium's properties. In contrast, time‐reflection remains more elusive due to the stringent requirement that the material parameters undergo a significant change within a timescale comparable to or shorter than a single period of the electromagnetic wave, demanding ultra‐fast switching capabilities.

In this context, Moussa et al. [77] experimentally demonstrated photonic time‐reflection and broadband frequency translation in a microwave transmission‐line metamaterial by implementing an abrupt, spatially uniform change in effective capacitance. This was achieved through a synchronized array of high‐speed voltage‐controlled switches, enabling the formation of sharp time interfaces essential for temporal wave manipulation. Further, Jones et al. [78] provided experimental evidence of phase‐conjugated time‐reflected waves in the microwave regime, revealing the signature time‐reversal symmetry properties at temporal boundaries. Using a microstrip transmission line with optically controlled, ultrafast impedance switching, they observed both time‐reflection and refraction from a 0.59 GHz pulse. Combining theoretical analysis and numerical simulations, the study reveals frequency translation and phase conjugation, with potential for enhanced effects in two spatial dimensions near the critical angle.

**FIGURE 3:**
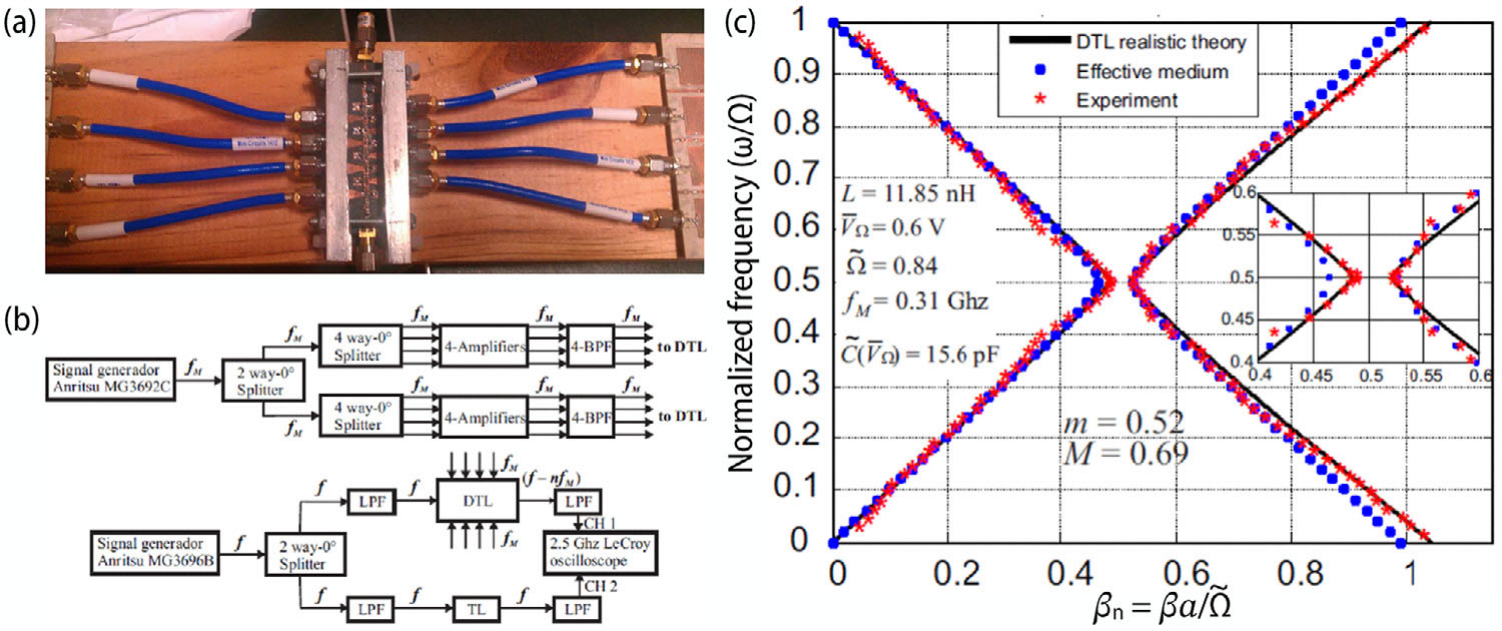
(a) Experimental setup of the fabricated eight‐cell dynamic transmission line (DTL), where the microstrip line is loaded with varactors. (b) Block diagram of the experimental setup. (c) Dispersion relations obtained theoretically (solid line), experimentally (asterisks), and from the effective medium model (dots), all showing the presence of a momentum bandgap in the band structure. Panels (a)–(c) are reproduced with permission [49]. Copyright 2016, IEEE.

Reyes‐Ayona and Halevi [49, 79] employed a dynamic transmission line (DTL) architecture to extract the photonic band structure of the system. They provided direct measurements of momentum bandgaps, along with theoretical calculations, thus offering an experimental demonstration of momentum‐resolved band formation in a periodically time‐modulated electromagnetic medium. Figure 3a shows the physical implementation of the DTL involving fabricating an eight‐cell DTL using microstrip technology, where each unit cell contained a varactor diode modulated by a synchronized 310 MHz AC voltage signal (see Figure 3b for the block diagram of the experimental setup). A separate signal generator produced probe waves of varying frequencies, which were split into two paths using a two‐way splitter. Using a high‐speed oscilloscope, phase shifts between the transmitted and reference signals were recorded and converted into a normalized phase constant (*β*). Repeating this procedure across a range of frequencies, they experimentally reconstructed the dispersion relation, revealing a clear bandgap in the momentum spectrum when the modulation strength exceeded a critical threshold (Figure 3c). This experimentally observed momentum gap closely matches with their theoretical predictions and illustrates the emergence of photonic bandgaps in time‐periodic media. These studies collectively offer steps in translating the abstract theoretical constructs of time‐varying photonics into experimentally accessible platforms within the microwave domain.

### 3.2 2D Materials and Metasurfaces

Realizing PTCs using 2D metasurfaces is perhaps easier at first glance than implementing them in bulk 3D materials, primarily because the required temporal modulation, such as surface conductivity or impedance, needs to be applied only at the surface rather than throughout the volume. This dimensional reduction may simplify experimental implementation by avoiding the complex task of achieving uniform time‐dependent modulation across a thick medium. To begin with, theoretical studies and simulations have demonstrated that time modulation of surface conductance or carrier density at timescales comparable to the plasmonic period is feasible in graphene, making it a promising candidate for implementing PTCs [80, 81, 82]. Kiselev et al. [83] showed a theoretical plasmonic analogue of a PTC, where amplitude‐modulated Floquet driving induces a temporal periodicity in the medium. The resulting parametric resonance opens a momentum bandgap in the plasmon dispersion, mirroring the band structure of PTC systems.

**FIGURE 4:**
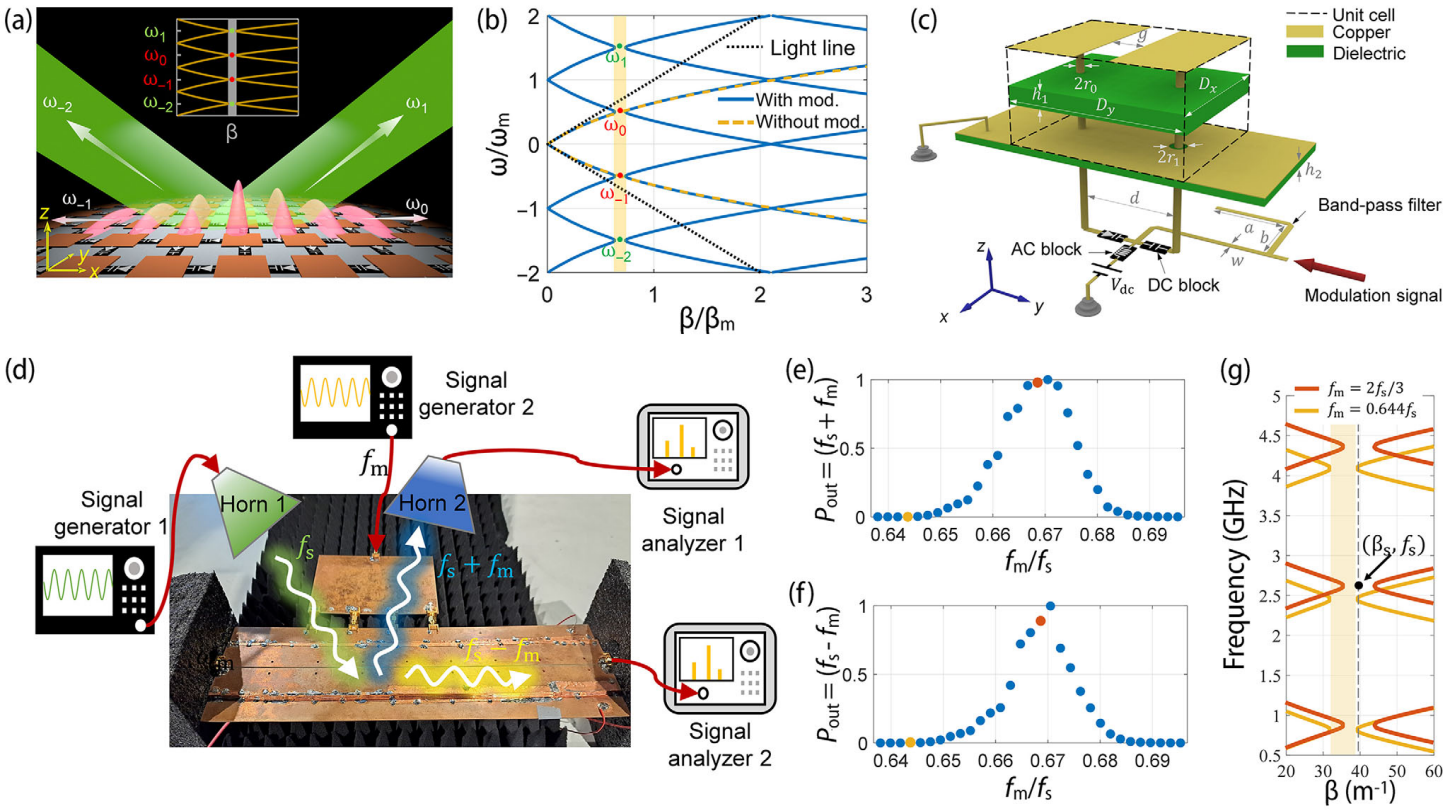
(a) Schematic illustration of a metasurface‐based PTC. A momentum bandgap (shaded in gray in the inset) emerges when the surface capacitance is modulated periodically in time. (b) Theoretical band structure of a time‐modulated capacitive boundary, showing the formation of a momentum bandgap (highlighted in orange). (c) Geometry of a unit cell (denoted in a dashed frame) used in the metasurface PTC, consisting of patterned metallic layers separated by dielectric substrates and periodically arranged in the *xy*‐plane. (d) Experimental configuration for probing the momentum bandgap via free‐space excitation using horn antennas, signal generators, and signal analyzers. (e–f) Experimentally measured and normalized power amplitudes of the generated harmonics at frequencies *f_s_* +*f_m_* and *f_s_*‐*f_m_*, respectively, as functions of normalized modulation frequency. (g) Extracted band structure from measurements, illustrating the momentum bandgap region. Panels (a–g) are reproduced with permission [55]. Copyright 2023, The American Association for the Advancement of Science.

Wang et al. [55] extended the concept of PTCs to a reduced‐dimensional platform by theoretically and numerically demonstrating that a time‐modulated metasurface can emulate the essential physics of bulk PTCs, including the emergence of momentum bandgaps under external excitation. They further validated this concept experimentally by implementing a metasurface‐based PTC at microwave frequencies and directly verifying the existence of a momentum gap. Figure 4a illustrates a conceptual schematic of a time‐modulated metasurface confined to the *xy*‐plane, composed of a spatially uniform but temporally varying capacitive surface. The surface capacitance is modulated periodically in time, effectively implementing a two‐dimensional PTC without any spatial periodicity. To investigate the resulting band structure, the authors performed an eigenmode analysis by solving Maxwell's equations under the influence of the time‐periodic boundary condition imposed by the metasurface. This analysis revealed the presence of a momentum bandgap, as shown in Figure 4b, characterized by the splitting of the photonic dispersion relation at specific values of in‐plane wavevector. Figure 4c shows the unit cell of the proposed experimental metasurface, which consists of a periodic array of metallic strips patterned in the *xy*‐plane on a grounded dielectric substrate. The top metallization comprises two parallel metal strips separated by a narrow gap, enabling capacitive coupling for transverse electric polarized surface waves. To achieve temporal modulation of the unit cell's effective capacitance, the strips are connected via vertical vias to a varactor diode embedded beneath the bottom metal layer. The full metasurface is composed of eight such unit cells arranged along the *x*‐direction, forming a finite structure for experimental characterization. In the experimental setup (Figure 4d), a directive horn antenna (Horn 1), driven by signal generator 1 at a signal frequency (*f_s_*) = 2613 MHz, launches an obliquely incident microwave beam onto the metasurface. A separate signal generator 2 provides a sinusoidal modulation signal frequency (*f_m_*), which modulates the capacitance of the varactors in time. The resulting scattered fields are measured using two signal analyzers, which selectively detect the lowest‐order scattered harmonics (*f_s_* +*f_m_* and *f_s_*‐*f_m_*), corresponding to free‐space and surface‐bound modes, respectively. By sweeping the *f_m_*, the power of induced harmonics was measured (Figure 4e,f). Finally, the corresponding band structure is computed numerically using the extracted time‐dependent effective capacitance of the metasurface, revealing the opening of a momentum bandgap (Figure 4g), confirmed by the suppression of the transmitted signal at certain in‐plane momenta.

### 3.3 Materials Responsive at Optical Frequencies: Challenges and Material Strategies

Across the various materials platforms considered, the temporally modulated parameters can be grouped into a few key categories. In PTC implementations at radio and microwave frequencies, the modulated quantity is typically the effective capacitance or impedance of a transmission line or metasurface unit cell, driven electronically through varactors or high‐speed switches. In 2D materials, the relevant parameter is the surface conductivity, which is modulated through ultrafast changes in carrier density induced optically or electrically. At optical frequencies, bulk or thin‐film materials rely on modulating their refractive index (*n*) or equivalently their dielectric permittivity (*ε*) at sub‐fs modulation speeds via mechanisms such as photoinduced phase transitions, ultrafast Kerr nonlinearities, or changes near the ENZ point in transparent conducting oxides (TCOs).

**FIGURE 5:**
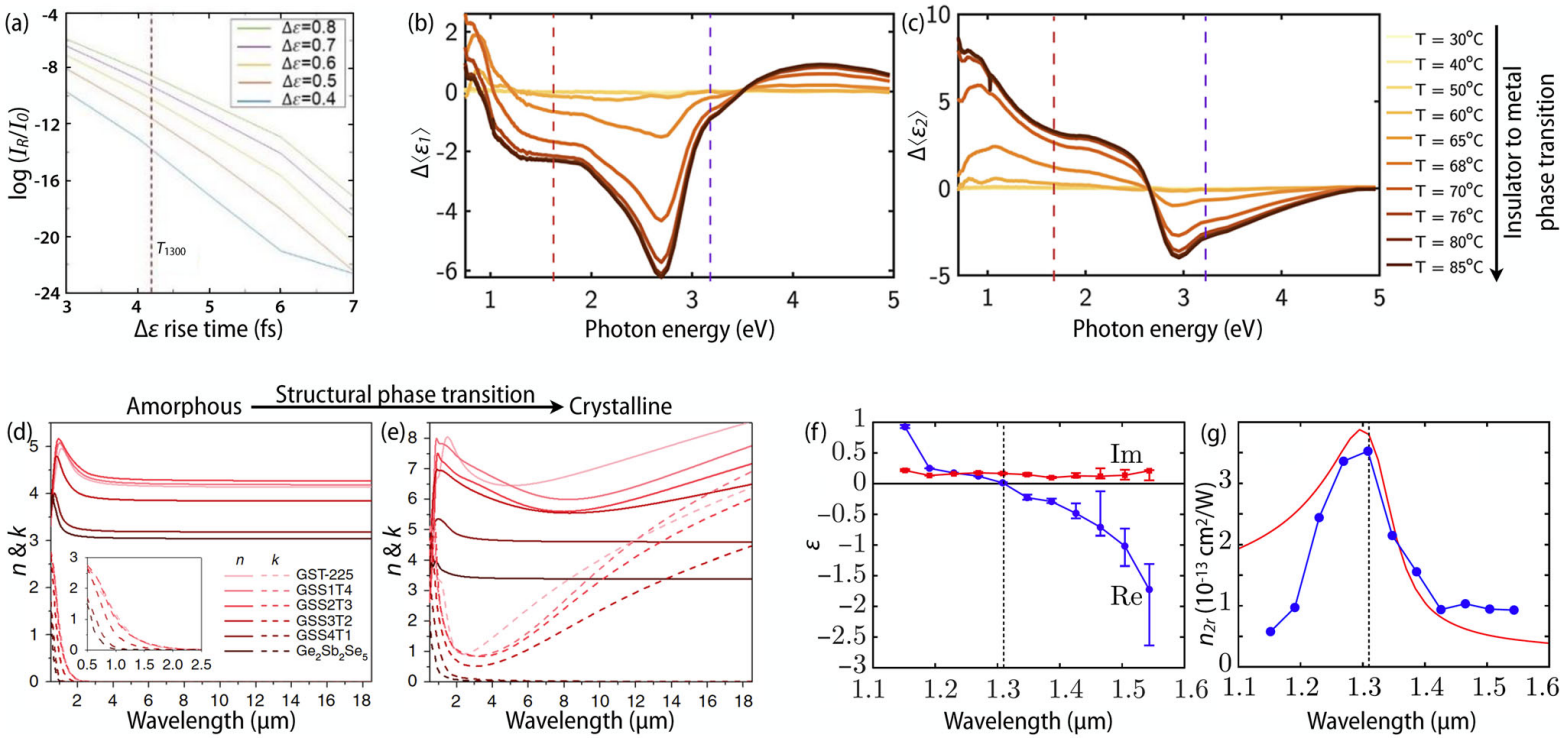
(a) Simulated time‐reflection coefficient for a light pulse at 1300 nm as a function of the dielectric permittivity change Δ*ε*, showing that time‐reflection is suppressed for modulation times slower than the optical cycle (4.3 fs). (b,c) Temperature‐dependent changes in the real and imaginary parts of the pseudodielectric function of a VO_2_ thin film, respectively. (d,e) Measured real (*n*) and imaginary (*k*) parts of the refractive index for the amorphous and crystalline phases of Ge_2_Sb_2_Se_x_Te_5‐x_ films, respectively. (f) Real and imaginary components of the linear permittivity, showing an ENZ wavelength near 1300 nm, and (g) real part of the nonlinear Kerr index, of an AZO thin film. Panel (a) is reproduced with permission [50]. Copyright 2021, Optica Publishing Group. Panels (b,c) are reproduced with permission [85]. Copyright 2024, American Chemical Society. Panels (d,e) are reproduced with permission [88]. Copyright 2019, Springer Nature. Panels (f,g) are reproduced with permission [51]. Copyright 2016, American Physical Society.

The experimental realization of PTCs in the optical frequency range remains a significant challenge. To date, no experimental demonstration has been achieved in this regime, though several theoretical proposals and proof‐of‐concept approaches have been reported. Two primary technical bottlenecks hinder realization: (a) achieving a sufficiently large optical modulation, as the relative width of the resulting momentum bandgap is directly proportional to the modulation strength, and (b) inducing such a modulation on ultrafast, ideally in sub‐femtosecond timescales. Temporal discontinuities capable of inducing time reflections have been demonstrated experimentally in macroscopic systems, such as water waves [84] and microwave transmission lines [55]. However, replicating such phenomena with optical frequency photons is substantially more difficult. Lustig et al. [50] demonstrated through numerical simulations that, in the optical regime, the time‐reflection coefficient decays exponentially when the refractive index (*n*) modulation is slower than a few femtoseconds and *n* < 1 (Figure 5a).

One class of materials considered for optical PTCs is phase‐change materials, which exhibit large changes in the optical properties such as refractive index (*n*), relative permittivity (*ε*) etc. across structural‐electronic phase transitions. In the proximity to the transition boundary, different phases can co‐exist and give rise to novel collective properties. For example, VO_2,_ as a phase transition material, undergoes a pronounced shift in its pseudodielectric function near the metal‐to‐insulator transition temperature (MIT). As shown by Gutiérrez et al. [85], both the real and imaginary components of the dielectric response vary strongly across the MIT (Figure 5b,c). A similar large modulation of permittivity and refractive index has also been observed in VO_2_ in the literature [86]. Further, Sood et al. [87] demonstrated that photoexcitation can trigger the monoclinic–rutile transition in VO_2_ on sub‐picosecond timescales (∼0.5–0.7 ps). Ge‐Sb‐Se‐Te alloys also show promise, with refractive index contrasts as large as Δ*n*≈2 in the mid‐infrared frequency [88]. Figure 5d,e shows the *n* and *k* values in the amorphous and crystalline phases, respectively, showing appreciable change for the Ge‐Sb‐Se‐Te system. Similarly, GeTe exhibits a rapid modulation of the real part of its dielectric function following excitation, with reported switching times below 250 fs [89].

These transitions have a thermal component, potentially posing a challenge for PTC operation. That is, the OFF to ON switching can be ultrafast, approaching femtosecond timescales, while the ON to OFF reversal might be thermally limited and is an avenue for further material engineering. Alternatively, Kerr‐type optical nonlinearities offer a path to achieve refractive index modulation on a fast timescale [46]. This third‐order nonlinearity enables instantaneous modulation of the refractive index by an intense pump field via the optical Kerr effect. The optical Kerr effect enables femtosecond‐scale refractive index changes in response to pump intensity. Hence, realizing a PTC requires rapid temporal modulation of the pump intensity to periodically vary the index. However, two critical limitations persist: (1) the nonlinear susceptibility is typically small in centrosymmetric material [90], and (2) periodic modulation of the pump intensity at optical timescales remains a challenge. Present systems are limited to modulation speeds in the GHz range, far below what is needed to match the optical carrier timescales [91].

Finally, we consider ENZ materials as promising candidates for realizing PTCs. ENZ materials are characterized by a dielectric permittivity that approaches zero near a characteristic frequency, typically close to their plasma frequency [92, 93]. Given the relation *n*∝√*ε* a small change in permittivity can induce large variations in *n*, when *ε* approaches to zero for ENZ materials [Δ*n* = (Δ *ε*)/2√*ε*)]. So, near the ENZ frequency, where the real part of *ε* passes through zero, and the imaginary part stays relatively small, these materials exhibit unconventional optical phenomena. TCOs, including cadmium oxide (CdO), indium tin oxide (ITO) and aluminum‐doped zinc oxide (AZO), are prototypical ENZ materials. Ultrafast pump‐probe studies on In‐doped CdO have shown that strong intraband excitation can induce large polarization switching on a sub‐picosecond timescale [94]. These silicon‐compatible photonic materials exhibit changes in dielectric permittivity on timescales significantly faster than those achievable through thermal, thermoelastic, or electro‐optic modulation mechanisms [51, 52, 95, 96].

**FIGURE 6:**
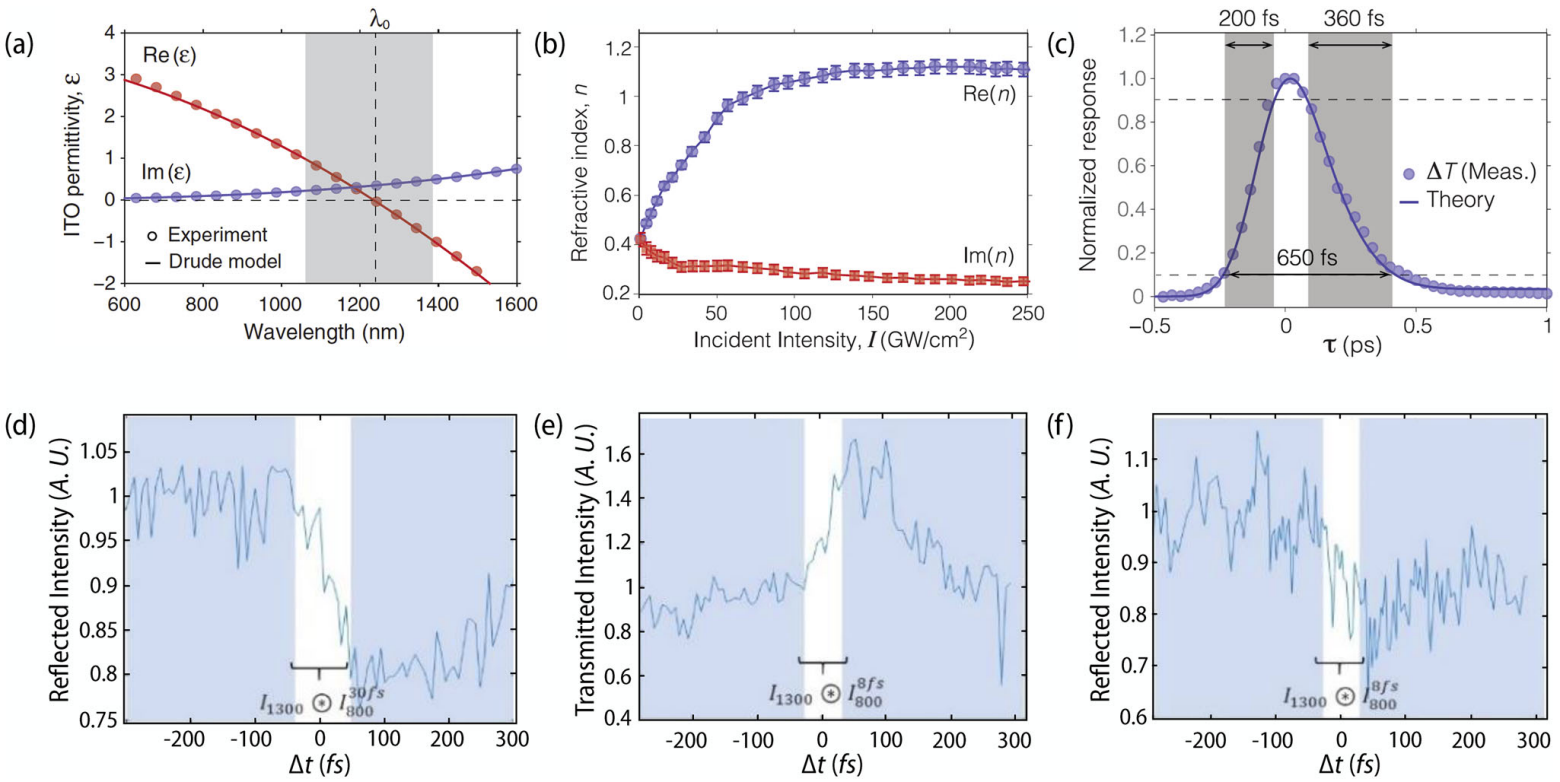
(a) Linear relative permittivity of an ITO film measured via spectroscopic ellipsometry, indicating an ENZ wavelength around 1240 nm. (b) Complex effective refractive index of the ITO film. (c) Time evolution of the normalized transient change in transmittance, measured using a degenerate pump‐probe setup, showing the characteristic rise and recovery times. (d) Reflected intensity from an AZO film as a function of time delay between a 30 fs pump and a 45 fs probe pulse. (e,f) Transmitted and reflected intensities from the AZO film under excitation by an 8 fs pump pulse. The narrower unshaded region corresponds to a shorter rise time. Panels (a–c) are reproduced with permission [52]. Copyright 2016, The American Association for the Advancement of Science. Panels (d–f) are reproduced with permission [50]. Copyright 2021, Optica Publishing Group.

Caspani et al. [51] identified the ENZ wavelength of an AZO film near 1300 nm (Figure 5f) and observed a sixfold enhancement in the Kerr nonlinear refractive index at this wavelength (Figure 5g), underscoring the significant field enhancement and strong light–matter interaction. Alam et al. [52] demonstrated that an ITO film exhibits an ENZ response at a wavelength of approximately 1240 nm, where the real part of the dielectric permittivity approaches zero (Figure 6a). At this ENZ wavelength, they reported a remarkable 170% modulation in the linear refractive index, accompanied by a pronounced third‐order optical nonlinearity (Figure 6b). The nonlinear response featured an ultrafast rise time of less than 200 fs and a recovery time of approximately 360 fs (Figure 6c), highlighting the potential of ITO for femtosecond‐scale optical modulation. A detailed summary of the nonlinear refractive indices across various materials is provided in ref. [97]. Time‐refraction effects in pump–probe experiments have also been observed in bulk ITO and AZO thin films [98, 99, 100, 101, 102]. Further studies on these materials demonstrated large, ultrafast refractive index modulations, with Δ*n* up to 0.5 in ITO and 0.15 in AZO occurring within 5–10 fs. Notably, ITO exhibits a fast relaxation time of 10–20 fs, making it an interesting candidate for realizing optical‐frequency photonic time crystals [103, 104]. Lustig et al. [50] experimentally demonstrated an ultrafast temporal boundary in the refractive index of AZO engineered with an ENZ point near 1500 nm, as a step toward realizing PTCs. Using a pump–probe setup with a 1300 nm probe and an 800 nm pump, they showed that a strong refractive index change (Δ*n*∼1) occurs on sub‐optical‐cycle timescales. Figure 6d shows the reflected signal with a 30 fs pump and 45 fs probe, where the material response is faster than both pulses. When the pump is compressed to 8 fs, the rise time shortens further, indicating an ultrafast response (Figure 6e,f). The measured decay time of the refractive index change is approximately 150 fs. This experimentally confirms that AZO can support femtosecond‐scale, order‐unity refractive index modulation, which is an essential requirement for time‐reflection, momentum bandgap formation, and ultimately, PTC behavior.

Further, various studies have also demonstrated that ultrafast modulation of ENZ properties can be realized in artificial ENZ platforms. In metal‐insulator‐metal nanocavities, optical pumping of a high‐energy ENZ mode produces a pronounced redshift of the low‐energy resonance, enabling picosecond‐scale all‐optical modulation [105]. CMOS‐compatible TiN/ITO multilayer ENZ metamaterials likewise support ultrafast all‐optical switching, showing few‐hundred‐femtosecond response times at two engineered ENZ wavelengths [106]. Pump‐probe measurements on Au/TiO_2_ ENZ multilayers show that femtosecond excitation generates hot electrons that induce large, transient changes in the effective permittivity by more than 400%. The relaxation occurs on a few‐picosecond timescale through electron‐phonon coupling, highlighting the strong ultrafast nonlinearity of ENZ multilayer systems [107]. Together, these works show that engineered ENZ architectures can support strong and ultrafast changes in their optical response, underscoring their potential relevance for time‐varying photonic systems.

In summary, while several material systems exhibit promising properties such as large refractive index contrast or ultrafast phase transitions, none yet simultaneously meet the stringent requirements of large Δ*n* and sub‐fs modulation speed necessary for realizing PTCs at optical frequencies. Continued efforts are required to identify or engineer materials and understand elementary interaction phenomena capable of ultrafast, high‐contrast refractive index switching under nonthermal or field‐driven mechanisms.

## 4 Conclusion and Outlook

Considerable progress has been made over the last few years in the realization of better modeling tools for PTCs and time‐varying metamaterials, alongside significant progress toward experimental realizations, particularly in the radiofrequency regime. This includes demonstrations of PTCs and the observation of exotic phenomena such as time‐reflection/refraction in the RF regime. While most RF experimental efforts highlighted in this perspective focused on dynamic transmission lines, nascent efforts toward other LTV devices, such as time‐varying metasurfaces, highlight the potential for novel materials in the RF domain. Of particular interest are next‐generation communication systems with applications in circulators, modulators, and even as highly reconfigurable antenna arrays. Experimental realizations will need to develop better controls for these LTV systems, due to the added dimensionality of temporal modulation. Further, modeling tools will need to be scaled to efficiently model full‐scale devices (i.e., antenna arrays). In addition, more accurate and efficient unit‐cell and device‐level modeling, which can treat the dispersive properties of LTV systems, and the complex waveforms associated with modern wireless communication schemes, are needed as well. At a more fundamental level, further exploration of more complex aperiodic modulation schemes alongside efforts to develop a deeper understanding of the physical bounds of LTV systems in the RF regime are necessary to better understand performance limitations and unlock their full potential.

**FIGURE 7:**
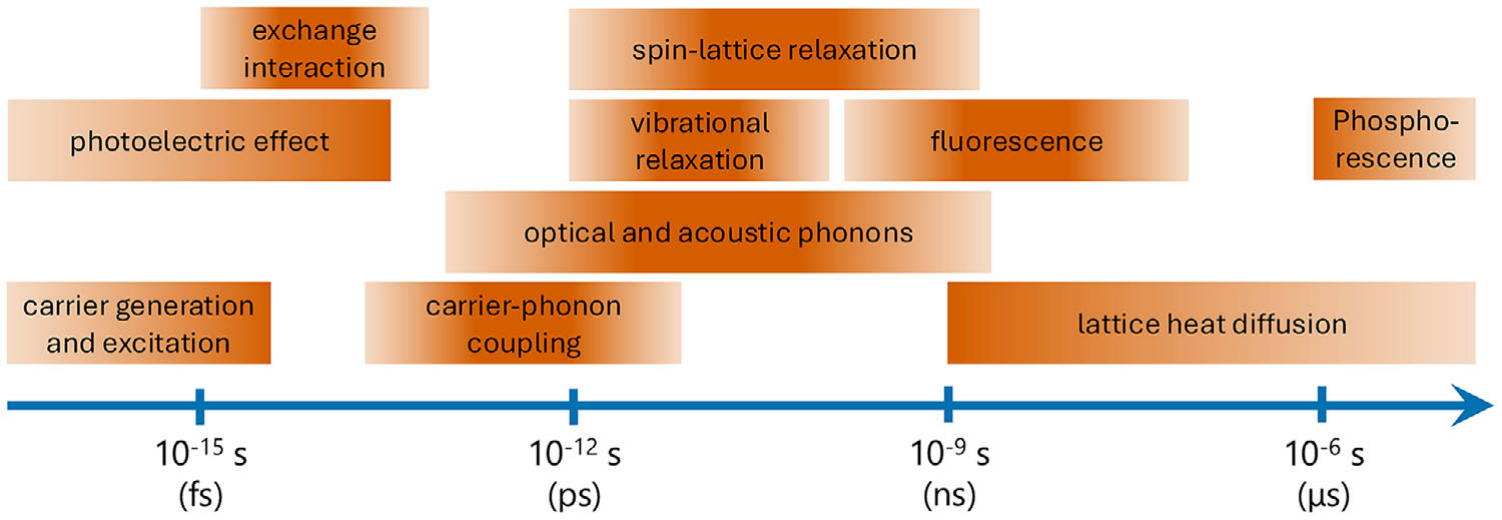
Representative timescales for different carrier dynamics processes. Electron‐photon interactions can occur on sub‐femtosecond timescales, while phonon processes may require tens of femtoseconds to picoseconds. In systems with spin order, it may be possible to realize novel coupling effects between photons and spins to realize ultrafast phenomena. Over slower timescales, heat gradients, mobile impurity drift, and other diffusional processes can become interesting knobs for designing photonic time metamatter.

In the optical regime, experimental progress has been more limited due to the immense material challenges in realizing large index modulation at ultrafast timescales, both in terms of the on/off modulation speed and periodicity of the modulation. Figure 7 summarizes various mechanisms involving radiation‐matter interactions with characteristic time scales. Clearly, electronic excitation suggests a routetowards femtosecond modulation timescales and must be coupled with spin order or non‐diffusive lattice re‐arrangements to obtain measurable changes to the optical constants. Depending on wavelengths of interest, other phenomena can become highly interesting as well, coupling electrons with phonons, or with mobile impurities etc. Hence, considerable research is needed to develop better tunable optical materials, both traditional and engineered materials, capable of large refractive index modulation at ultrafast timescales, both in terms of the on/off time and periodicity of the modulation. Beyond fundamental materials research, continued efforts to engineer devices with novel modulation approaches are desirable. Particularly, implementation schemes of PTCs and time‐varying metamaterials, which relax the constraint on the ultrafast time scale of periodic modulation, will open new frontiers. Here, further efforts toward optical isolators, modulators, and other device architectures that only require periodic modulation on GHz time scales seem primed for continued exploration.

Overall, the study of PTCs and time‐varying materials remains a rich area of study across science and engineering disciplines. To unlock the full potential of this field, increasingly collaborative efforts are needed to bring together physicists, chemists, material scientists, and electrical engineers. Such interdisciplinary efforts are essential to develop better optical and RF materials capable of ultrafast, large index modulation, which can then be leveraged to explore novel phenomena and device architectures of time‐varying electromagnetic systems.

## Conflicts of Interest

The authors declare no conflicts of interest.
